# Vulpeculin: a novel and abundant lipocalin in the urine of the common brushtail possum, *Trichosurus vulpecula*

**DOI:** 10.1098/rsob.200218

**Published:** 2020-10-07

**Authors:** Grace M. Loxley, David O. Hooks, Aristotelis Antonopoulos, Anne Dell, Stuart M. Haslam, Wayne L. Linklater, Jane L. Hurst, Robert J. Beynon

**Affiliations:** 1 Centre for Proteome Research, Institute of Systems, Molecular and Integrative Biology, University of Liverpool, Liverpool L69 7ZB, UK; 2 Centre for Biodiversity and Restoration Ecology, School of Biological Sciences, Victoria University of Wellington, Wellington 6140, New Zealand; 3 Department of Life Sciences, Imperial College London, London SW7 2AZ, UK; 4 Department of Environmental Studies, California State University, Sacramento, CA 95819, USA; 5 Mammalian Behaviour and Evolution Group, Institute of Infection, Veterinary and Ecological Sciences, University of Liverpool, Leahurst Campus, Neston CH64 7TE, UK

**Keywords:** brushtail possum, *Trichosurus vulpecula*, major urinary protein, metabolic labelling, mass spectrometry

## Abstract

Lipocalins are a family of secreted proteins. They are capable of binding small lipophilic compounds and have been extensively studied for their role in chemosignalling in rodent urine. Urine of the common brushtail possum (*Trichosurus vulpecula*) contains a prominent glycoprotein of 20 kDa, expressed in both sexes. We have isolated this protein and determined its primary sequence by mass spectrometry, including the use of metabolic labelling to resolve the leucine/isoleucine isobaric ambiguity. The protein sequence was identified as a lipocalin, and phylogenetic analysis grouped the protein with other marsupial lipocalin sequences in a phylogenetic clade distinct from established cross-species lipocalin sub-families. The pattern of expression in possum urine and the similarity in sequence and structure to other lipocalins suggests this protein may have a role in brushtail possum chemosignalling.

## 1. Introduction

The common brushtail possum, *Trichosurus vulpecula* [1] is a marsupial native to Australia where it is a protected wildlife species in mainland states [2]. In some urban and agricultural regions, however, possums reach high densities and population management is required; an annual cull is permitted to protect agricultural crops in Tasmania, for example [3]. Since its introduction to New Zealand in the mid-nineteenth century to develop a fur industry [4], the possum has been reclassified as a national pest species. There, it is a reservoir and vector for bovine tuberculosis (*Mycobacterium bovis*), taking an economic toll on the cattle and deer industry [5–7]. An arboreal omnivore, mostly browsing, its feeding depredates native flora and fauna [8–13]. Currently, the species is one of five introduced predators that are the subject of a nationwide eradication effort by New Zealand's government. Advanced control tools, such as fertility control, super-lures and automated killing devices, face a knowledge gap with regard to the behaviour and physiology of marsupial species in general, and the brushtail possum specifically. In particular, our understanding of possum reproductive and social signalling, and how they communicate and coordinate on the landscape, is rudimentary.

Possums are generally solitary-living animals with small, overlapping home ranges [14,15], meeting physically during the breeding season, especially if their ranges overlap [16]. Males' ranges are larger than females', within which they move more frequently, and male dominance is correlated with age and size [17]. Under favourable conditions, females can raise a single pouched young twice a year [18] and have a lifespan of approximately 13 years [19]. The primary breeding season takes place in autumn but breeding behaviour appears to be flexible and dependent on environment [20].

Possums exhibit social behaviours observed in many other mammals, involving social hierarchy, territorial defence, individual recognition and mating [17]. Dominant males, and occasionally dominant females, exhibit scent marking behaviour including chinning, chesting, cloacal dragging, urine dribbling and face-washing [17,21,22]. The purpose of these behaviours has yet to be established, as does the physiological and molecular mechanism behind them. Although no pheromones have been identified to date, they have an extensive olfactory epithelium and a well-developed vomeronasal organ, both of which indicate an advanced olfactory capability [17]. The sternal patch is particularly distinctive in sexually mature males and is due to the presence of active apocrine and sebaceous glands that secrete waxy lipids [23]. The cloacal and paracloacal glands, arranged in pairs on either side of the cloaca, produce either an oily secretion or cell-containing medium [24–26]. While the presence of these glands and secretory components is largely established, no correlations have been made with behavioural traits. In addition to glandular secretions, cell-containing material is continuously secreted into urine [25], making urine a potential source of possum chemosignalling.

Urine-mediated chemosignalling is evident across *Mammalia*. In the house mouse, urine is used to convey sex, age, reproductive status, hierarchy and individuality [27–35]. The complexity of this signal is reliant on the major urinary proteins (MUPs), a subfamily of lipocalin proteins that are secreted into urine [32–35]. MUP genes have been identified across the mammalian lineage [36], but are not the only lipocalin subfamily involved with scent signalling; other lipocalin subfamilies expressed in saliva [37–39] and vaginal discharge [40] have also been associated with a behavioural response. The highly conserved structure of the lipocalin family of proteins lends itself to olfactory signalling. As eight-stranded anti-parallel β-barrels, they are firstly stable in the environment, prolonging a signal, and secondly have an affinity for volatile hydrophobic ligands within the internal calyx [41,42]. Understanding of protein-mediated chemical signalling has only recently come under scrutiny. However, there are increasing examples of an individual protein class, often a lipocalin, present at high levels in mammalian scent secretions [40,43–51]. Often accompanied with sexually dimorphic or seasonal expression under hormonal control, it is considered an indication of a chemosignalling function.

Little is known about putative chemosignalling proteins in marsupials, although a well-developed vomeronasal organ similar to rodents [52–55] suggests a potential for detection of large chemosignalling molecules that might include proteins. Lipocalin-like proteins are excreted in milk during lactation of the Tamar wallaby [56–58], red kangaroo [59], grey kangaroo [59], quokka [60] and the brushtail possum [61], some of which are homologous to marsupial β-lactoglobulins [59,61], and another more closely resembles rodent MUPs [61]. Trichosurin (a MUP-like lipocalin) was the first solved 3D structure of a lipocalin from a metatherian [62] and has been conjectured to bind small phenolic compounds that might prime the neonate liver to produce enzymes required to metabolize toxic plant phenols, reducing dietary limitations [62]. In this study, we have characterized the protein content of the urine of brushtail possum to explore the potential role of proteins in possum urine-mediated chemosignalling. We were able to characterize a glycosylated lipocalin that has the potential to act as a binding protein for volatile pheromones.

## 2. Methods

### 2.1. Urine sampling

Urine samples were collected from wild-caught *T. vulpecula* captured in the Wellington and Canterbury Provinces, New Zealand. Possums were kept in separate wooden hutches (1.5 × 1.5 × 2.5 m), provided with pelleted dry, domestic stock food (Reliance) *ad libitum*, and given fruit and vegetables every second day, and freshly cut browse weekly. Urine was collected by removing each possum from its sleeping sack or box and restraining it, whereupon most immediately urinated. Possums were released after approximately 30 s, regardless of whether or not they urinated. The urine was collected in 50 ml tubes and stored frozen (−80°C) until analysis. Some urine samples were freeze-dried prior to shipping to the UK for mass spectrometric analysis.

### 2.2. SDS-PAGE analysis

Urinary proteins were initially analysed by SDS-PAGE, using a Tris-chloride/Tris glycine discontinuous buffer system under reducing conditions [63]. Electrophoresis was conducted in a mini-protean system (Bio-Rad, Hemel Hempstead, UK) and separated proteins were visualized with Coomassie blue.

### 2.3. Protein and creatinine assays

Urine dilution was assessed by the total creatinine concentration of each sample, measured using the alkaline picrate assay kit from Sigma-Aldrich (Sigma, Poole, UK). Total protein concentration of each sample was calculated using the Coomassie Plus protein assay kit (Pierce, Rockford, USA).

### 2.4. Anion exchange chromatography

Urinary proteins were separated by anion exchange chromatography. Male urine samples were pooled and dialysed into 20 mM Tris-HCl pH 8.5 using 3.5 MWCO SnakeSkin dialysis tubing (Thermo Fisher Scientific) overnight. Buffer was then replaced, and dialysis was performed for a further four hours. Particulates were cleared by centrifugation at 2000*g*, the supernatant was retained and the protein content measured. Protein (350 µg) was then loaded onto a 1 ml RESOURCE Q column (GE Life Sciences) and eluted at 1 ml min^−1^ over a linear gradient of 0–0.8 M NaCl in 20 mM Tris-HCl buffer using an ÄKTA purifier (GE Healthcare). Absorbance at 280 nm was monitored and fractions were collected manually, and analysed by SDS-PAGE. Fractions containing the 25 kDa protein were pooled for further analysis.

### 2.5. Intact protein mass measurement

Urine samples or protein from anion exchange fractions (concentrated, see below) were diluted in acetonitrile (5% (v/v)) and formic acid (0.1% (v/v)) in HPLC grade water. Protein (0.1 ng male samples, 1.6 ng female samples) was injected onto a C4 desalting trap (Waters MassPREP Micro desalting column, 2.1 × 5 mm, 20 µm particle size, 1000 Å pore size) (Waters, Manchester, UK) that was fitted on a Waters nano ACQUITY Ultra Performance liquid chromatography (UPLC) system. The chromatography system was coupled to a Waters SYNAPT G2-Si QTOF mass spectrometer fitted with an electrospray source. Protein eluted over a 10 min acetonitrile gradient (5–95% (v/v)) at 40 µl min^−1^. Data were collected between *m/z* 500–3000. The data were processed using maximum entropy deconvolution (MAX ENT 1, Mass Lynx version 4.1, Waters) at 1 Da/channel over a mass range of 15 000–25 000 Da. The mass spectrometer was calibrated externally with horse heart myoglobin (250 fmol µl^−1^, Sigma).

### 2.6. In-gel digestion

Protein bands from SDS-PAGE were digested with trypsin to generate peptides suitable for further analysis by MALDI-ToF mass spectrometry and LC-MS/MS. Excised gel plugs (approximately 1 mm^2^) were destained with 50% (v/v) 50 mM NH_4_HCO_3_/MeCN for 15 min at 37°C. They were then reduced in 10 mM dithiothreitol at 60°C for 30 min and carbamidomethylated in 55 mM iodoacetamide for 45 min at room temperature. The gel plug was washed with 25 mM NH_4_HCO_3_ for 15 min at 37°C before dehydration with 50 µl MeCN for 15 min at 37°C. Excess acetonitrile was removed and the gel plug was rehydrated with sequencing grade trypsin (Roche, Lewes, UK) to approximately 50 : 1 protein : enzyme ratio in 25 mM NH_4_HCO_3_ and incubated overnight.

### 2.7. Peptide analysis by MALDI-ToF MS

Peptides were analysed by MALDI-TOF mass spectrometry to acquire the masses of peptides derived from band-specific proteins. MALDI-TOF spectra were acquired using a Bruker ultrafleXtreme mass spectrometer in reflectron mode. Samples were mixed with MALDI matrix (saturated solution of α-cyano-4-hydroxycinnamic acid in 50% (v/v) ACN/0.2% (v/v) TFA) in a 1 : 1 ratio and spotted onto a target plate before being left to air dry. The laser frequency was 1000 Hz, laser energy 30% of maximum and 2000 laser shots were collected per spectrum, between 700–4000 *m/z*. All aspects of data acquisition and machine management were controlled through the flexControl, and data processing was performed in flexAnalysis (Bruker Daltonics).

### 2.8. Protein desalting and concentration

For digestion with multiple proteases, pooled protein-containing anion exchange fractions were made up to 1 ml with milli-Q water and concentrated on 10 µl Strataclean resin (Agilent, Santa Clara, US) in the following manner. After the addition of the beads, samples were vortex mixed at 2000 r.p.m. for 1 min and centrifuged at 2000 r.p.m. for 1 min. The supernatant was removed and 1 ml milliQ water was added, followed by vortex mixing and centrifugation as above. This wash step was repeated once more and the protein concentrated on the resin was made up to digestion volume with the appropriate buffer. Prior to deglycosylation, the pooled protein-containing fractions were concentrated up to 10× using Vivaspin columns (Sartorius), by centrifugation at 13 000*g*. To desalt, water was added up to the original volume and the sample was again centrifuged to concentrate the protein.

### 2.9. In-solution digestion

Purified protein (2 µg) was diluted in 25 mM NH_4_HCO_3_ and incubated with 0.05% (final concentration) Rapigest SF Sufactant (Waters, Manchester, UK) at 80°C for 10 min. To reduce disulfide bonds, DTT in 25 mM NH_4_HCO_3_ was added to a final concentration of 10 mM and incubated at 60°C for 10 min. The sample was then carbamidomethylated with iodoacetamide (3 mM final concentration) in 25 mM NH_4_HCO_3_ and incubated at room temperature for 30 min in the dark. The protein was then digested at 37°C overnight by addition of sequencing grade trypsin or endopeptidase Glu-C (Roche, Lewes, UK) in 25 mM NH_4_HCO_3_ to a protein : enzyme ratio of 50 : 1. Digestion with endopeptidase Lys-C (Roche, Lewes, UK) followed a similar protocol using 25 mM Tris HCl, 1 mM EDTA pH 8.5 as the digestion buffer. Digestion with Asp-N (Sigma-Aldrich, UK) was performed in 50 mM AmBic pH 8.0 with a protein : enzyme ratio of 20 : 1.

### 2.10. Peptide analysis by LC-MS/MS

To collect data at high resolution, samples were analysed using an Ultimate 3000 UHPLC system (Dionex/Thermo Fisher Scientific, Hemel Hempstead, UK) coupled to a Q Exactive mass spectrometer (Thermo Fisher Scientific, Hemel Hempstead, UK). Peptides (500 fmol) were loaded onto a trap column (Acclaim PepMap 100, 2 cm × 75 µm inner diameter, C18, 3 µm, 100 Å) at 5 µl min^−1^ with an aqueous solution containing 0.1% (v/v) TFA and 2% (v/v) acetonitrile. After 3 min, the trap column was set in-line with an analytical column (Easy-Spray PepMap RSLC 50 cm × 75 µm inner diameter, C18, 2 µm, 100 Å) (Dionex). Peptides were eluted by using an appropriate mixture of solvents A and B. Solvent A was HPLC grade water with 0.1% (v/v) formic acid, and solvent B was HPLC grade acetonitrile 80% (v/v) with 0.1% (v/v) formic acid. Separations were performed by applying a linear gradient of 3.8% to 50% solvent B over 35 min at 300 nl min^−1^ followed by a washing step (5 min at 99% solvent B) and an equilibration step (15 min at 3.8% solvent B). The mass spectrometer was operated in data dependent positive (ESI+) mode to automatically switch between full scan MS and MS/MS acquisition. Survey full scan MS spectra (*m/z* 300–2000) were acquired in the Orbitrap with 70 000 resolution (at 200 *m/z*) after accumulation of ions to 1 × 10^6^ target value based on predictive automatic gain control (AGC) values from the previous full scan. Dynamic exclusion was set to 20 s. The 10 most intense multiply charged ions (*z* ≥ 2) were sequentially isolated and fragmented in the octopole collision cell by higher energy collisional dissociation (HCD) with a fixed injection time of 120 ms and 35 000 resolution. The mass spectrometer was calibrated using a ready to use positive ion calibration solution from the instrument manufacturer (Thermo Fisher Scientific, Hemel Hempstead, UK). The solution contains a mixture of caffeine, MRFA, Ultramark 1621, and n-butylamine in an acetonitrile : methanol : water solution containing acetic acid (1% v/v). The mass spectrometer conditions were as follows: spray voltage, 1.9 kV, no sheath or auxiliary gas flow; heated capillary temperature, 250°C; normalized HCD collision energy 30%. The MS/MS ion selection threshold was set to 1 × 10^4^ counts and a 2 Th isolation width was set.

### 2.11. Sequencing *de novo*

Data was analysed in PEAKS (Bioinformatics Solutions Inc.). Precursor masses were corrected and sequences were generated *de novo* within the following parameters: precursor error tolerance ±10 ppm, fragment ion error tolerance ±0.01 Da and up to five candidates per spectrum were reported. A fixed post-translational modification (PTM) of carbamidomethylated cysteine residues and variable modifications of oxidated methionine residues were specified. Abundant, unidentified peptides sequenced *de novo* by the software, with an average local confidence (ALC) score of greater than 55% and a mass tag cut-off score of 50%, were aligned using overlapping sections with assistance from multiple sequence alignments of homologous proteins.

### 2.12. Database searching

Database searches were performed under the same error tolerance and modification parameters. No non-specific cleavages were allowed in primary PEAKS DB searches, and the maximum number of missed cleavages was set to three. Additional variable modifications were added when appropriate. Deamidation of asparagine was added for analysis of peptides deglycosylated using PNGase F, and trideuterium-labelled leucine was added to assist with identification of heavy leucine residues. Identifications were made from PEAKS SPIDER searches to allow for predicted mutations for improved identification. The data were searched against all mammalian sequences in SwissProt, and all *Monodelphis domestica* sequences in UniProt.

### 2.13. Protein deglycosylation

Protein was deglycosylated according to NEB Protocol. Purified protein (0.5 µg) was made up to 10 µl and incubated with 2 µl glycoprotein deglycosylation buffer for 10 min at 100°C. The protein sample was cooled on ice before mixing with 1 µl 10% NP-40, 1 µl 10× GlycoBuffer and 1 µl PNGase F (glycerol-free) (New England Biosciences). After incubation at 37°C for 1 h, 10 µl deglycosylation mixture was analysed by SDS-PAGE. To deglycosylate the protein prior to in-solution digestion, 2 µg purified protein was made up to 17 µl in HPLC-grade H_2_O, denatured by the addition of 1 µl 1% w/v Rapigest and incubated at 80°C for 10 min. The sample was then reduced by the addition of 1 µl 10 mM dithiothreitol and incubating for 10 min at 60°C. PNGase F, 1 µl, was then added and the reaction mixture was incubated at 37°C for 1 h. The reaction mixture was then expanded to 45 µl in 25 mM NH_4_HCO_3_ and the protein was carbamidomethylated by the addition of 2.5 µl of 33 mg ml^−1^ iodoacetamide at room temperature for 30 min before proteolytic digestion. To deglycosylate prior to analysis of intact proteins, 300 pmol purified protein was incubated in 5 M urea at 37°C for 1 h. DTT was added to a final concentration of 50 mM and the sample was incubated for a further 30 min at 37°C. PNGase F (500 units) was added and the sample was incubated for 1 h at 37°C. Buffer exchange was performed using Zeba spin desalting columns (7 K MWCO) (Thermo Fisher Scientific, Hemel Hempstead, UK) and the deglycosylated protein (10 pmol) was subsequently analysed by LC-MS.

### 2.14. Glycomic analysis

MALDI-MS based glycomic analysis was as described previously [64]. Briefly, each sample was reduced in 4 M guanidine-HCl (Pierce, Cramlington, Northumberland, UK) followed by carboxymethylation, and trypsin digestion. The digested glycoprotein was then purified by HLB plus Sep-Pak (Waters Corp, Hertfordshire, UK; 186000132). *N*-glycans were released by peptide *N*-glycosidase F (E.C. 3.5.1.52; Roche Applied Science, Burgess Hill, UK) digestion. Released *N*-glycans were permethylated using the sodium hydroxide procedure and purified by classic C_18_-Sep-Pak (Waters, WAT051910). Permethylated *N*-glycans were eluted at the 50% acetonitrile fraction.

MS and MS/MS data were acquired using a 4800 MALDI-TοF/TοF mass spectrometer (Applied Biosystems, Darmstadt, Germany). Permethylated samples were dissolved in 10 µl of methanol and 1 µl of dissolved sample was premixed with 1 µl of matrix (10 mg ml^−1^ 3,4-diaminobenzophenone in 75% (v/v) aqueous acetonitrile), spotted onto a target plate and dried under vacuum. For the MS/MS studies the collision energy was set to 1 kV, and argon was used as the collision gas. The 4700 Calibration standard kit, calmix (Applied Biosystems), was used as the external calibrant for the MS mode and [Glu1] fibrinopeptide B human (Sigma-Aldrich) was used as an external calibrant for the MS/MS mode. MS and MS/MS data were processed using Data Explorer 4.9 Software (Applied Biosystems). The processed spectra were subjected to manual assignment and annotation with the aid of a glycobioinformatics tool, GlycoWorkBench [65]. The proposed assignments for the selected peaks were based on ^12^C isotopic composition together with knowledge of the relevant biosynthetic pathways. The proposed structures were then confirmed by data obtained from MS/MS.

### 2.15. Discrimination of leucine and isoleucine by metabolic labelling

Isobaric amino acids leucine and isoleucine were discriminated by the addition of L-leucine-5,5,5-d_3_ to the diet (as a supplement to mashed banana) of one possum over a 7 day feeding period [47]. Urine was collected once daily and the remaining food removed and weighed. The added L-leucine-5,5,5-d_3_ comprised approximately 40% of the dietary leucine this possum received over the feeding period. Urine samples were desalted using Zeba spin columns (7 K MWCO) (Thermo Scientific) before in-solution digestion, as described above. Samples from each day of the feeding period were digested with trypsin only to monitor heavy leucine incorporation. The desalted urine sample from the final day of heavy leucine incorporation was deglycosylated prior to proteolytic digestion with either trypsin only, Glu-C only, or with both Asp-N and trypsin, whereby trypsin was added four hours after Asp-N, followed by overnight incubation as above. Peptides were analysed by tandem mass spectrometry as described above.

### 2.16. BLAST searching

Whole protein sequences and shorter peptide sequences were searched using BLAST against all non-redundant protein sequences in the NCBI database (https://blast.ncbi.nlm.nih.gov/Blast.cgi) or UniProt (https://www.uniprot.org/blast/). For searching of full protein sequences, the blastp algorithm was employed, with the following parameters. The maximum target sequences was set to 100, the expect threshold was set to 10, the word size was set to 6 and the maximum matches in a query range was set to 0. The scoring matrix employed was BLOSUM62, and gap costs were specified to Existence: 11 Extension: 1, with a conditional compositional score matrix adjustment. For analysis of peptide sequences, search parameters were adjusted automatically to account for short sequence lengths, as follows; expect threshold was 200 000, word size was 2, the matrix used was PAM30 and no compositional adjustment was made.

### 2.17. Multiple sequence alignment

Sequences selected for multiple sequence alignment were first analysed by the SignalP server [66] to identify signal peptides which were removed prior to further analyses. Clustal Omega (https://www.ebi.ac.uk/Tools/msa/clustalo/) [67] was employed under default parameters. JalView [68] was used to visualize the alignment and alignments were coloured according to percentage identity.

### 2.18. Phylogenetic analysis

The finished protein sequence was searched using BLAST against all marsupial protein sequences in NCBI database (taxID: 9263) or UniProt. Significant (*e*-value < 0.01) results were included in the analysis. Mammalian proteins belonging to the lipocalin family, previously identified as having a putative chemosignalling function, were also included. A full list of UniProt and GenBank accession numbers for the protein sequences used can be found in electronic supplementary material, table S1. Predicted signal peptides were removed using the SignalP 4.1 server [66] and aligned using Clustal Omega [67]. The resulting alignment was viewed in JalView [68] and MEGA 6.06 [69] was used for evolutionary analyses. The evolutionary history was inferred by using the maximum-likelihood method based on the JTT matrix-based model. Bootstrapping analysis using 500 replicates was carried out and the tree with the highest log likelihood (−16 237.8530) is shown. Branches corresponding to partitions reproduced in less than 50% bootstrap replicates were collapsed. All positions containing site coverage of less than 95% were eliminated and the resulting 131 positions were analysed.

### 2.19. Homology modelling

All RCSB Protein Data Bank (PDB) structures were searched against the newly sequenced protein using BLAST (https://blast.ncbi.nlm.nih.gov/Blast.cgi). The six top-scoring sequences were aligned with vulpeculin using Clustal Omega [67] and the corresponding structures used as templates. The alignments were manually adjusted to fit the structural information given in the .pdb file, and 10 models were generated based on each template using Modeller 9.16 [70]. Model quality was assessed using MolProbity [71] and QMEAN score [72]. The highest quality model was viewed and annotated in PyMOL [73].

### 2.20. Statistical analysis

Linear mixed-effects models for analysis of protein and creatinine data was performed in RStudio v. 1.1.463 [74] using the package lme4 [75] which uses a restricted maximum-likelihood (REML) approach. All other statistical analyses were performed in SPSS Statistics v. 24 (IBM Corp., 2016).

## 3. Results

### 3.1. Expression and heterogeneity of protein in possum urine

**Figure 1.**
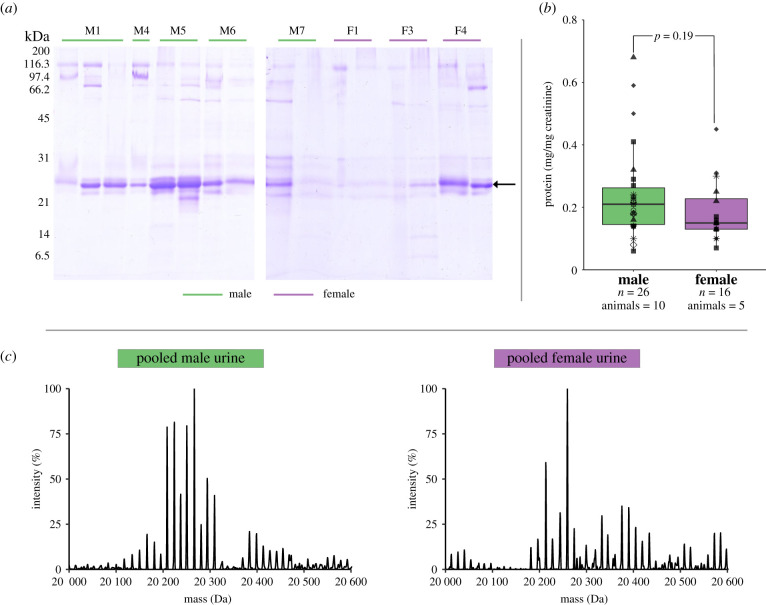
Analysis of proteins in possum urine. (*a*) Urinary proteins, equivalent to 20 µl of each urine sample, were analysed by SDS-PAGE. (*b*) Urinary protein output was assessed as protein : creatine ratio (individual animals are differentiated by symbol type). (*c*) The masses of intact proteins from male urine and female urine were analysed by LC-MS and revealed complexity within the 20.0–20.6 kDa region (spectra from pooled male urine and pooled female urine).

Urine samples were collected from 10 male (*n* = 26 samples) and five female (*n* = 16 samples) common brushtail possums. As an initial assessment of protein complexity, SDS-PAGE analysis revealed a predominant cluster of protein bands resolving in the region of 20–25 kDa (figure 1*a*) that varied in intensity between samples. Overall urinary protein output, expressed as µg protein per µg creatinine to correct for urine dilution [76], did not differ significantly between males (0.24 ± 0.03 mg protein (mg creatinine)^−1^) and females (0.19 ± 0.02 mg protein (mg creatinine)^−1^, *t*_40_ = 1.35, *p* = 0.19, figure 1*b*). There was also no difference in urinary protein output between samples collected during breeding (spring, autumn: 0.24 ± 0.04, *n* = 8) and non-breeding seasons (summer, winter: 0.22 ± 0.02, *n* = 34; *t*_40_ = 0.41, *p* = 0.68). The dilution of urine samples, assessed by creatinine concentration, did not differ between the sexes (*t*_40_ = 0.039, *p* = 0.97) or between seasons (*t*_40_ = −0.66, *p* = 0.51). To obtain further information on protein complexity, urine samples were desalted and proteins analysed intact by LC-MS. Complex spectra were observed for each sample, with a range of abundant masses predominantly between 20.0–20.6 kDa, with dominant adjacent peaks commonly separated by 42 Da, interspersed with peaks separated by 14 Da (pooled urine, figure 1*c*; electronic supplementary material, figure S1).

To investigate the potential for peptide-level heterogeneity, the main bands resolving at approximately 25 kDa were excised from SDS-PAGE gels and digested in-gel using trypsin and analysed by MALDI-ToF mass spectrometry. The resulting peptide mass fingerprints (PMF) (electronic supplementary material, figure S2) were highly similar from all individuals of both sexes, suggesting that a common protein component was present in each individual, regardless of sex.

### 3.2. Protein sequencing of the novel possum urinary protein

To investigate the nature of the urinary protein complement further, urinary proteins from seven male and five female possums were individually digested with trypsin and analysed by LC-MS/MS. Tandem mass spectrometry data were searched against databases comprising all mammalian proteins in SwissProt, or all protein sequences from *Monodelphis domestica* (grey short-tailed opossum) in UniProt, but no highly scoring protein matches were obtained. Thus, an iterative approach was adopted to support protein discovery. First, the highly abundant, confident peptide masses also observed in the MALDI-ToF MS-generated PMFs were selected for direct targeted sequencing *de novo* in PEAKS software. Then, the predicted sequences of these precursor ions, with masses matching the high intensity peaks in the PMF, were in turn searched individually against mammalian sequences using BLAST [77], optimized for short sequence probes. The recurrent highly-scoring match for these abundant peptides was the trichosurin-like protein from *M. domestica* (NCBI Reference Sequence: XP_007475413.1 or UniProt accession: F7F0X2). Eight peptides sequenced *de novo* covered 21% of the homologous protein at 55–73% identity. Finally, the trichosurin-like protein from *M. domestica* was subsequently searched against all mammalian protein sequences in the NCBI database using BLAST. A multiple sequence alignment was generated from the top 25 scoring hits using MUSCLE [78] and used as a scaffold to support further protein sequencing (electronic supplementary material, figure S3).

To gain further information about urinary protein complexity, urinary proteins were further resolved by anion exchange chromatography. Male or female urine samples were pooled and dialysed against 20 mM Tris-HCl, pH 8.5, after which 350 µg protein was resolved by anion exchange chromatography at pH 8.5 with a salt gradient from 0 to 1.0 M NaCl. Protein peaks with high absorbance at 280 nm were collected manually and SDS-PAGE was used to identify the elution position of the 20 kDa protein(s). LC-MS analysis of intact proteins confirmed that the three mass peaks previously observed in whole urine co-eluted at the same NaCl concentration in the gradient (electronic supplementary material, figure S4). Fractions containing these proteins were pooled and concentrated prior to further sequence analysis.

The 20 kDa protein mixture from anion exchange chromatography was digested with trypsin, endopeptidase Lys-C, endopeptidase Glu-C or endopeptidase Asp-N to generate independent sets of peptides that were analysed by LC-MS/MS and sequenced using PEAKS. High-quality sequences obtained *de novo* were used to inform and assist the manual assembly of the protein sequence. Peptide sequence data from all LC-MS/MS runs were re-searched against the newly constructed protein sequence in PEAKS to assist further sequencing *de novo*. Through this iterative process, a confident protein sequence was recovered (electronic supplementary material, figure S5), apart from the inability to discriminate between the isobaric amino acids leucine and isoleucine (denoted here as ‘J’). Further, a gap of approximately 13 amino acids near the N-terminus differentiated the sequenced protein to aligned homologous proteins (electronic supplementary material, figure S5). While this could be a genuine *T. vulpeculus* sequence deletion, the alignment revealed that this region would have contained the fully conserved lipocalin motif G-X-W [41]. Additionally, the calculated average mass of the protein, as manually sequenced, was 17 267 Da, whereas the measured average masses of the three protein species were 20 252 Da, 20 294 Da and 20 335 Da; thus the predicted mass of the protein sequence obtained was smaller than that of the observed proteins by approximately 3 kDa. Examination of homologous protein sequences suggested that this was unlikely to be covered solely by a missing segment of the sequence, raising the additional possibility of glycosylation. Indeed, this was also supported by the heterogeneity in the intact masses obtained for this protein, probably separated by 42 Da, and consistent with a variable degree of acetylation, probably on the glycan.

**Figure 2.**
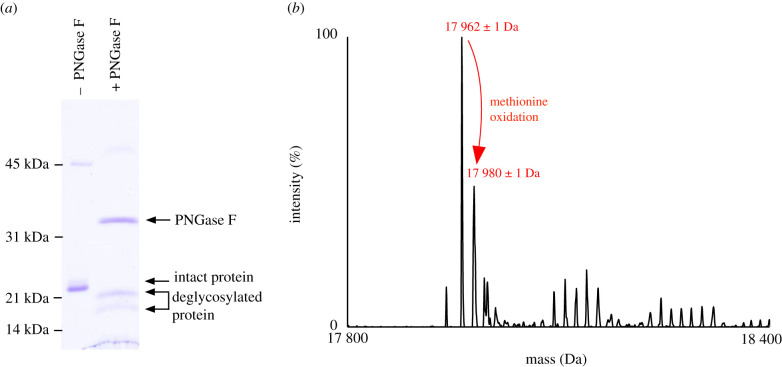
Deglycosylation of the possum urinary protein. (*a*) Pooled male urine was incubated with PNGase F and analysed by SDS-PAGE, in which an increased mobility was observed in the enzyme-treated sample. (*b*) This reduction in molecular weight was confirmed by intact mass analysis, in which a single predominant peak of 17 962 Da was observed.

To investigate the possibility of glycosylation, pooled male urine was incubated with PNGase F to remove N-linked glycans. The resulting reaction mixture was analysed by SDS-PAGE and a mobility shift was observed, consistent with an effective deglycosylation reaction (figure 2*a*). The deglycosylation protocol was adapted to be compatible with LC-MS, and analysis of the intact protein confirmed that after treatment, two predominant protein peaks of masses 17 962 and 17 980 Da, were observed (figure 2*b*), over 2 kDa smaller than the parent protein. The mass difference between these peaks is synonymous with an oxidation modification that occurs commonly on methionine residues.

A deglycosylation step using PNGase F was incorporated into the in-solution digestion protocol, and the deglycosylated protein was then digested with either trypsin, endopeptidase Glu-C or endopeptidase Lys-C and again analysed by LC-MS/MS.

**Figure 3.**
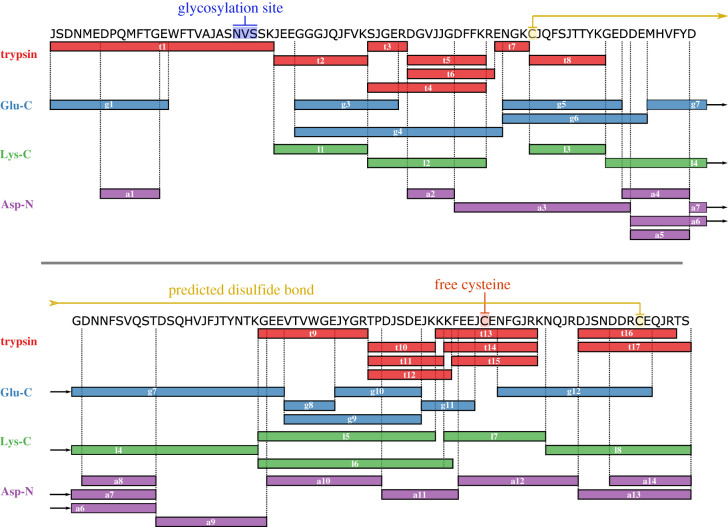
Complete protein sequencing of the possum urinary protein. The purified protein from pooled male urine was digested with trypsin (t1…t19), Glu-C (g1…g12), Lys-C (l1…l8) and Asp-N (a1…a13) and sequenced *de novo* with the assistance of PEAKS (Bioinformatics Solutions Inc.). The N-terminus was sequenced from deglycosylated peptides. Overlapping peptides were aligned to form a sequence coverage map that shared homology with other closely-related lipocalin sequences.

Sequencing of the deglycosylated protein, determined from fragment ion spectra (electronic supplementary material, figure S6), confirmed the previously acquired sequence and revealed the remainder of the protein sequence at the N-terminus. The tryptic peptide JSDNMEDPQMFTGEWFTVAJAS[**N>D**]VSSK contained the conserved glycosylation motif N-X-[S/T], where the asparagine residue had been converted to an aspartic acid [**N>D**] by the action of PNGase F. Further, this sequence contained the conserved lipocalin motif G-X-W. It was sequenced from the fragment ion spectrum generated from the [M + 3H]^3+^ precursor ion of 1002.45 *m/z* (figure 3, t1), in addition to the fragment ion spectra from the modified peptide, oxidized at one or both of the methionine residues present within this peptide (1007.79 *m/z* and 1013.12 *m/z*, respectively). This sequence was confirmed by the Glu-C peptides JSDNMEDPQMFTGE, with one missed cleavage, and WFTVAJAS[N>D]VSSKJEE. The latter peptide overlapped the tryptic peptide JEEGGGJQJFVK, identified from fragment ion spectra generated from the [M + 2H]^2+^ precursor ion of 645.36 *m/z*. This was combined with previous sequencing efforts, until overlapping peptides were obtained for the whole protein (figure 3). The structural stability of lipocalins is assisted by the presence of disulfide bonds. The major urinary proteins contain three conserved cysteine residues at C_64_, C_138_ and C_157_ (mature sequence numbering system), of which C_64_ and C_157_ are oxidized to form a disulfide bond. The newly sequenced possum protein also contains three cysteine residues at C_60_, C_132_ and C_151_, that, when aligned with other homologous proteins including mouse MUPs, align perfectly (figure 6). We therefore hypothesize that the C_60_ and C_151_ residues also oxidize to generate a disulfide bond.

The total mass predicted for the protein sequence (17 962 Da) matched the mass measured after deglycosylation (17 963 ± 1 Da), allowing for the 1 Da increase in mass originating from the conversion of glycosylated asparagine to deglycosylated aspartic acid. To confirm if the protein was the same in females, protein from pooled urine samples from female brushtail possums were also subject to chromatographic separation, analysis of the intact and deglycosylated protein by LC-MS, and analysis of the sequence by proteolytic cleavage of both glycosylated and deglycosylated protein with multiple enzymes to generate overlapping peptides. Good sequence coverage was observed across the whole novel protein sequence, and the deglycosylated mass observed was consonant with data observed for the male samples. We can therefore conclude that the protein component for this novel protein is identical in both males and females.

### 3.3. Resolution of isobaric residue ambiguity

**Figure 4.**
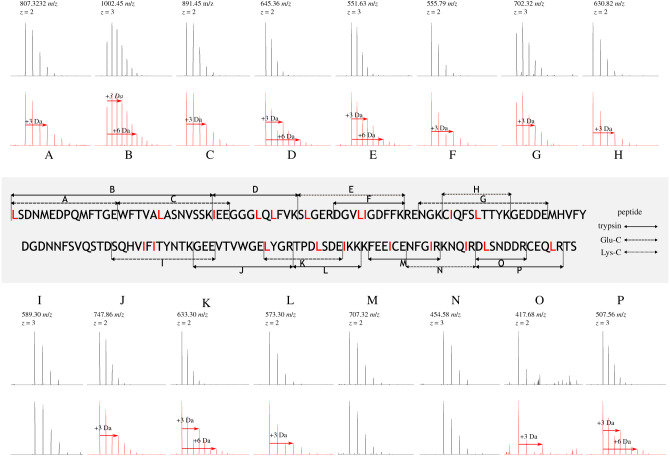
Assignment of leucine/isoleucine ambiguities by stable isotope labelling. Isotope-labelled 5,5,5-d3 leucine was incorporated into the diet of a single male brushtail possum during which time urine was collected daily. Urine samples were subsequently digested in-solution with trypsin, Glu-C or Lys-C (solid, dashed and dotted lines, respectively) to obtain sequence coverage of all Leu/Ile-containing peptides, and leucine sites were assigned according to a +3 Da increase in precursor ion spectra (A–P). Predicted leucine and isoleucine sites were confirmed with fragment ion spectra.

To complete the sequence, we resolved the ambiguity of the isobaric residues leucine and isoleucine using metabolic labelling. Isotope-labelled leucine, 5,5,5-[^2^H] (‘heavy’) leucine was added to the diet of a male possum over the course of 6 days. Urine samples were collected prior to incorporation, daily throughout the dietary labelling and for 2 days once the normal diet was resumed. Samples from each day were digested in-solution with trypsin to monitor heavy leucine incorporation (electronic supplementary material, figure S7). Heavy-labelled leucine reached a maximum on day 3 of dietary supplementation at 13 ± 0.5 (mean ± s.e.) % and the urine sample from day 3 was deglycosylated and digested in-solution with trypsin, endopeptidase Glu-C or endopeptidase Lys-C to provide complete resolution of Leu/Ile-ambiguities. The isobaric residues were determined twofold. Firstly, by identifying a +3 Da shift in precursor ion spectra and secondly, by locating the introduction of a +3 Da peak in the **b**- or **y**-ion series of product ion spectra. The incorporation rate was calculated using tryptic peptides containing one instance of leucine. Nonlinear optimization was used to model the difference between the experimental isotope pattern and a theoretical isotopic spectrum generated from a combination of a non-labelled peptide and a 5,5,5-[^2^H] labelled peptide. Confident resolution of putative leucine and isoleucine sites within peptides was obtained from a combination of precursor and fragment ion spectra (figure 4).

### 3.4. Phylogenetic analysis

Distinction of the isobaric residues leucine and isoleucine allowed in-depth exploration of sequence homology to the novel protein in *Mammalia*, to suggest potential protein function. Marsupial proteins for sequence comparison (*n* = 31) were identified by searching the finished protein sequence using BLAST against all marsupial non-redundant protein sequences in the NCBI database (taxID: 9263) or all marsupial sequences in UniProt. For comparison, representatives from lipocalin sub-families with members previously identified as having a putative chemosignalling function were included, all of which are from placental mammals. These include MUPs, salivary lipocalins, epididymal-specific lipocalins and odorant binding proteins (OBPs). UniProt and GenBank accession numbers for the final 114 protein sequences used can be found in electronic supplementary material, table S1. Predicted signal peptides were removed using the SignalP 4.1 server [66] and the remaining sequences aligned using Clustal Omega [67]. The resulting alignment was viewed in JalView [68] (electronic supplementary material, figure S8) and MEGA 6.06 [69] was used for phylogenetic analyses.

**Figure 5.**
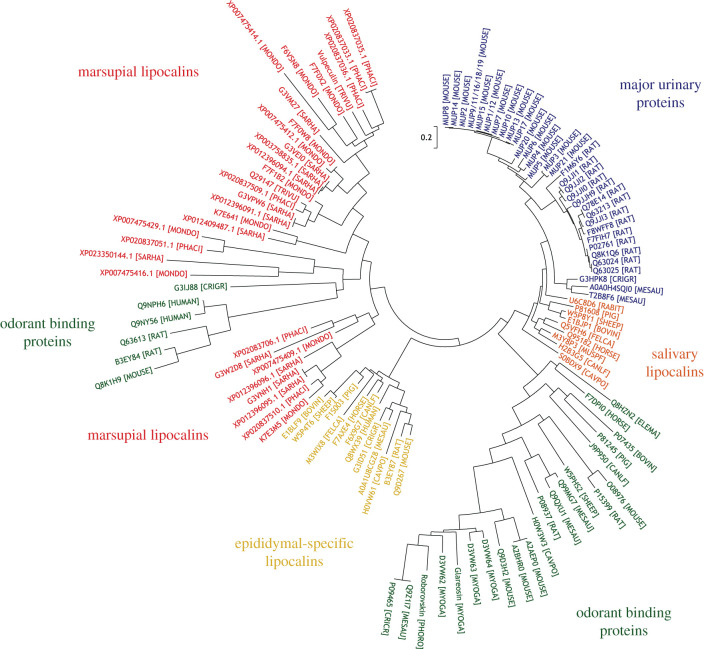
Phylogenetic analysis. The evolutionary history was inferred by using the maximum-likelihood method based on the Whelan & Goldman model [79]. The tree with the highest log likelihood (−16 237.8530) is shown. Initial tree(s) for the heuristic search were obtained automatically by applying Neighbor-Join and BioNJ algorithms to a matrix of pairwise distances estimated using a JTT model, and then selecting the topology with superior log likelihood value. A discrete gamma distribution was used to model evolutionary rate differences among sites (five categories (+*G*, parameter = 6.8547)). The tree is drawn to scale, with branch lengths measured in the number of substitutions per site. The analysis involved 114 amino acid sequences. All positions with less than 95% site coverage were eliminated. That is, fewer than 5% alignment gaps, missing data, and ambiguous bases were allowed at any position. There were 131 positions in the final dataset. Evolutionary analyses were conducted in MEGA6 [69]. The location of vulpeculin is highlighted with an arrow.

The resulting phylogenetic tree has a number of notable features (figure 5). The sequences from placental mammals are arranged within their established lipocalin classes. There is clear separation between MUP sequences from the Norway rat and the house mouse, the salivary lipocalin sequences and the epididymal-specific lipocalins. One major clade containing only odorant binding proteins is also observed, although six OBP sequences are located elsewhere in the phylogenetic tree. The marsupial lipocalins (displayed in red), however, group as two distinct clades. One smaller group containing eight sequences are displayed as more evolutionarily distant than the remaining 24. Within these 24 remaining marsupial sequences, four are grouped with the subset OBP sequence outliers. However, the long branch lengths within this clade suggest that this group of sequences is evolutionarily distant within the group. The remaining 20 marsupial sequences are situated in a clade with close branch lengths, and include the novel protein sequence (indicated), in addition to the trichosurin-like protein from *M. domestica* (UniProt accession F7F0X2) which was used to inform initial sequencing efforts, and trichosurin (UniProt accession no. Q29147).

The division between marsupial lipocalins and those from placental mammals suggests that the marsupial lineage diverged prior to the separation into the distinct lipocalin classes we see. Consequently, marsupial lipocalins could contain a distinct class of lipocalin, or distinct classes, within those analysed. The naming of the marsupial lipocalins, the majority of which derive from unannotated genomes, makes this distinction unclear. Many are denoted as ‘trichosurin-like’, ‘major urinary protein-like’ or indicate homology to the other remaining placental mammal lipocalin classes examined. However, as these marsupial lipocalins are clearly distinct from already established lipocalin sub-families, it would be appropriate to classify them as such. However, protein sequences from only four marsupials were available: the brushtail possum (*T. vulpecula*), the grey short-tailed opossum (*M. domestica*), the Tasmanian devil (*Sarcophilus harrisii*) and the koala (*Phascolarcturus cinereus*), from incomplete and unannotated genomes. Due to the knowledge gap in marsupial genomics, distinction of further lipocalin classes within marsupial species is unclear, and it was decided not to implement trichosurin in the name of the novel protein. We propose that the *T. vulpecula* protein that has been discovered and fully sequenced here be referred to as ‘vulpeculin’.

### 3.5. Homology modelling of vulpeculin

While the primary structure of lipocalins can vary significantly, the tertiary structure is highly conserved, forming an eight-stranded anti-parallel beta-barrel surrounding an internal calyx that often has ligand-binding properties [41]. To investigate if vulpeculin shares the same conserved structure, the sequence was subject to structural homology modelling to generate a 3D structure.

**Figure 6.**
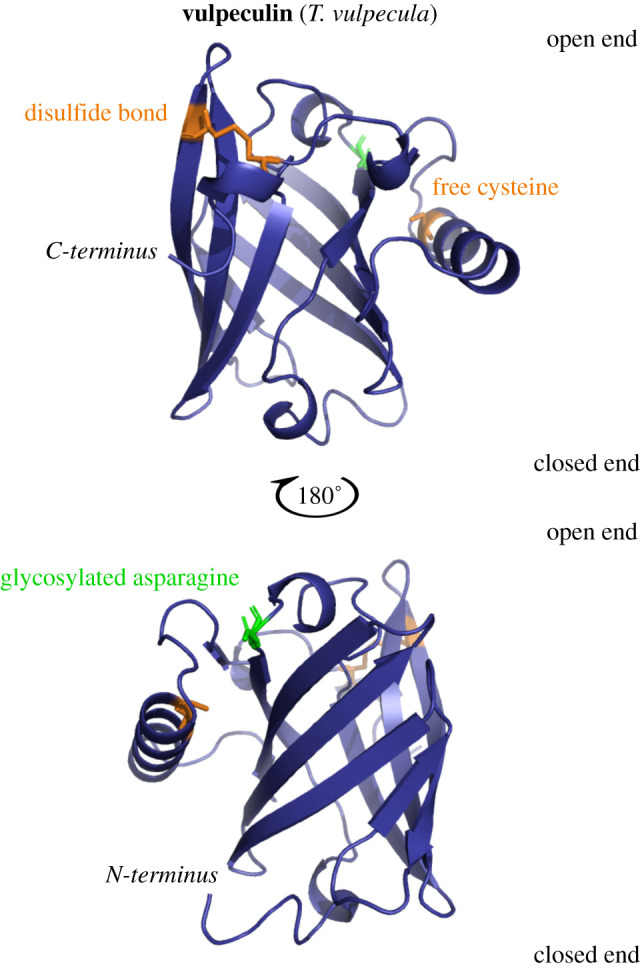
Structural homology modelling of vulpeculin. The completed vulpeculin was modelled using homologous templates. Homologous structures were identified in the RCSB Protein Data Bank using BLAST. Sequences were aligned in Clustal Omega [67] and models were generated in Modeller 9.16 [70]. Ten models were produced per template, and predicted structures were validated using the QMEAN server [72] and MolProbity [71]. The most confident model was built based on the structure of trichosurin (*T. vulpecula*), experimentally determined by X-ray crystallography.

Vulpeculin was modelled against the RCSB Protein Data Bank (PDB) structure of trichosurin (2R73), that had been experimentally determined by X-ray crystallography [62] using Modeller 9.16 [70] and model quality was assessed using Ramachandran assessment in MolProbity [71] and QMEAN score [72]. The model of vulpeculin was viewed and annotated in PyMOL [73]. A conserved beta-barrel structure is observed, consistent with its classification as a lipocalin (figure 6). The glycosylation site (green) is situated at the ‘open’ end according to lipocalin structure terminology [41]. All residues that make up the alpha-helices and beta-sheets were high scoring in the model, indicating these regions are strongly conserved, and as expected, the loops were less confidently modelled. The attached glycan could not be modelled but given the location at the ‘open’ end of the lipocalin, it may play a role in binding specificity of ligands accessing the internal calyx.

### 3.6. Preliminary glycan characterization

**Figure 7.**
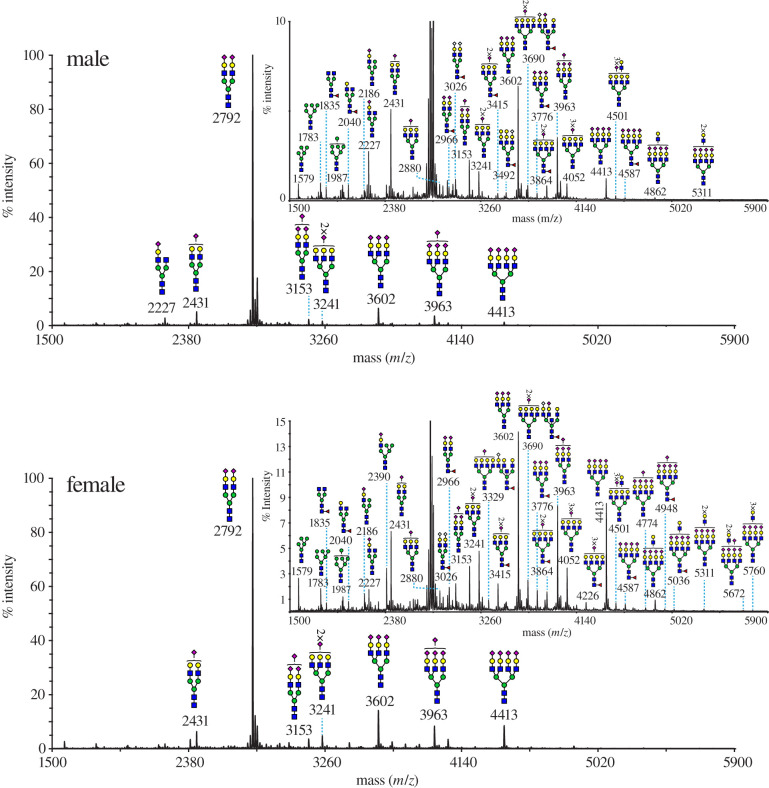
Glycan analysis of vulpeculin. MALDI-ToF MS of permethylated N-glycans from male and female possum urinary vulpeculin was used to reveal the glycan structure. N-glycans are from the 50% MeCN fraction (see Methods). Insets are range expanded spectra in the *y*-axis (% intensity) from 0–10 and or 0–15% of the original *y*-axis for the male and female sample respectively. All structures are [M + Na]^+^. Structures above a bracket were not unequivocally defined. Putative structures are based on composition, tandem MS and knowledge of biosynthetic pathways.

Having established that vulpeculin is a glycoprotein, we performed a preliminary analysis on the glycans attached to the protein. MALDI-ToF MS and MS/MS were employed for the characterization of the N-glycans from the vulpeculin proteins. The N-glycans from both male and female protein had very similar structural features (figure 7). The major species corresponded to non-core fucosylated N-glycans ranging from bi- to tetra-antennary structures capped with NeuAc residues (*m/z* 2431, 2792, 3602, 3963 and 4413). In addition, minor components were detected (figure 7, insets) that consisted of high mannose (*m/z* 1579, 1783 and 1987), hybrid (*m/z* 2186) and complex structures which were either non-core fucosylated (*m/z* 2880, 3690, 4052 and 4862), or core fucosylated (*m/z* 2040, 2966, 3415, 3776 and 3864). Complex structures had LacNAc antenna extensions and were capped mainly with NeuAc and to a lesser extent with NeuGc residues (*m/z* 2227, and 3026), or Gal-α1,3-Gal epitopes (*m/z* 3492) (figure 7, insets).

## 4. Discussion

We describe a novel glycosylated protein, vulpeculin, as the most abundant protein present in urine of the male and female brushtail possum, *Trichosurus vulpecula*. The protein component was sequenced *de novo*, including discrimination of leucine and isoleucine residues using metabolic labelling. Vulpeculin was identified as a lipocalin, sharing the highly conserved beta-barrel structure of this protein class. Phylogenetic analysis classifies vulpeculin within the same clade as other marsupial lipocalins, distinct from the established lipocalin sub-families of placental mammals. Analysis of the separated glycan indicated similar carbohydrate structures in both male and female samples, however a match could not be made with the masses observed from LC-MS analysis of the intact glycoprotein.

Although the genome sequence for *T. vulpecula* is unavailable, investigation into the location of genes encoding proteins from other marsupials reveals a cluster of lipocalin-encoding genes. A species-specific expansion of both vomeronasal-2 receptor genes and MUP-like genes has previously been reported in the grey short-tailed opossum, *M. domestica*, from which parallels are drawn to the corresponding expansions in mice and rats [32]. However, it has been noted that again, these sequences were sufficiently divergent from placental mammal MUPs to suggest classification within an alternative lipocalin class [36]. A similar grouping of marsupial lipocalins, distinct from other mammalian sequences is also paralleled in β-lactoglobulins [61]. The cluster of lipocalin genes on chromosome 1 of the *M. domestica* genome encodes at least six lipocalin proteins (UniProt accessions: F6VSN8, F7F0X2, F7F1B2, F7F0W8, K7E641, all included in the analysis, and K7E3M5, not identified by BLAST searching so not included). Less published information is available for *S. harrisii* (Tasmanian devil) and *P. cinereus* (koala), although tBLASTn searching of the vulpeculin protein sequence identified a number of lipocalin-encoding genes located together in *S. harrisii* (encoding for G3VM27, G3VPW6, G3VEI0 (UniProt accession), XP_012396096.1 (NCBI accession), and G3VNH1, denoted here as XP_012396095.1) and in *P. cinereus* (encoding for XP_020837033.1, XP_020837035.1, XP_020837036.1, XP_20837509.1 and XP_020837510.1, NCBI accession). This suggests these proteins may be co-expressed, and it may be the case that some level of polymorphism exists. However, while the expansion is considerably more than many mammalian species, the homology between each of these proteins is not comparable to that of the MUPs. The lack of genome data for *T. vulpecula* means that it is not possible to tell if this potential polymorphism also exists in the brushtail possum from genome data, and where it might be expressed, if so. The only evidence for closely related protein sequences is that of trichosurin, expressed during lactation of *T. vulpecula*.

Intact mass analysis determined that the dominant protein in the majority of samples had an intact mass between 20 and 21 kDa. However, the disparity in masses determined between samples has still not been fully resolved. The repeated 42 Da increments are derived from modification of the glycan (acetylation is the most likely explanation), or from different variants of the glycan itself. While the protein component of this molecule is unchanging between individuals, the attached glycan is yet to be fully explored as a source of heterogeneity.

Homology analysis of the completed protein sequence provided considerable insight into evolutionary relationships within the lipocalin protein family. Phylogenetic analysis revealed that marsupial lipocalins identified as homologous to the novel protein did not cluster into the well-described lipocalin families of eutherian (placental) mammals. As the described protein was situated within a cluster of marsupial lipocalins, we propose the name vulpeculin, avoiding the implication of a close similarity to other mammalian lipocalin families. Our interest in urinary proteins with a potential involvement in chemosignalling focused our phylogenetic analysis on other lipocalin classes known to have such roles. However, further analysis of a more expansive range of lipocalins, including lipocalins identified in marsupial milk (for example, trichosurin), may provide further insight into the divergence of this class of marsupial lipocalins. The closest inferred homology was to other marsupial sequences identified using BLAST. Although the genome sequence for *T. vulpecula* is unavailable, investigation into the location of genes encoding proteins from other marsupials reveals a cluster of lipocalin-encoding genes. A species-specific expansion of both vomeronasal-2 receptor genes and MUP-like genes has previously been reported in the grey short-tailed opossum, *M. domestica*, from which parallels are drawn to the corresponding expansions in the mouse and the rat [32]. However, these sequences were sufficiently divergent from placental mammal MUPs to suggest classification within an alternative lipocalin class [36], and the same grouping of marsupial lipocalins, distinct from other mammalian sequences is also paralleled in β-lactoglobulins [61]. The cluster of lipocalin genes on chromosome 1 of the *M. domestica* genome encodes at least six lipocalin proteins (UniProt accessions: F6VSN8, F7F0X2, F7F1B2, F7F0W8, K7E641, all included in the analysis, and K7E3M5, not identified by BLAST searching so not included). Less published information is available for *S. harrisii* (Tasmanian devil) and *P. cinereus* (koala), although tBLASTn searching of the vulpeculin protein sequence identified a number of lipocalin-encoding genes located together in *S. harrisii* (encoding for G3VM27, G3VPW6, G3VEI0 (UniProt accession), XP_012396096.1 (NCBI accession), and G3VNH1, denoted here as XP_012396095.1) and in *P. cinereus* (encoding for XP_020837033.1, XP_020837035.1, XP_020837036.1, XP_20837509.1 and XP_020837510.1, NCBI accession).

The strongest evidence for protein sequences homologous to vulpeculin is of trichosurin, expressed during lactation of *T. vulpecula*. There is some suggestion that trichosurin is glycosylated; the same disparity is observed between SDS-PAGE resolved molecular weight and the size of the sequenced peptide [61], and automatic assignment in UniProt indicates two putative N-glycosylation sites at residues 67 and 148 [80]. The identification of MUP-like lipocalins in urine and milk suggests that this considerable investment in protein output may have a role in chemosignalling. It also highlights a potentially important role of glycosylation, which, since post-translational modifications are not template-driven, can be difficult to compare between and within species on a large scale. Finally, we did not consider the ligand binding capability of vulpeculin. As the majority of samples were freeze dried for transportation, the low-molecular-weight component of volatile urine samples could not be investigated.

## Data accessibility

This article has no additional data.
